# Astaxanthin Mitigates Dextran Sulfate Sodium-Induced Colitis in Mice by Repairing the Intestinal Barrier, Regulating Specific Intestinal Flora, and Reducing Inflammatory Cell Infiltration

**DOI:** 10.1155/jimr/3381950

**Published:** 2025-08-21

**Authors:** Guangzhe Shen, Du Yu, Longfei Xu, Tiezheng Yang

**Affiliations:** Anorectal Department, First Affiliated Hospital of Changchun University of Traditional Chinese Medicine, Changchun 130021, China

**Keywords:** astaxanthin (Ax), colitis, DSS, intestinal barrier, intestinal flora

## Abstract

As a dietary supplement for humans and animals, Astaxanthin (Ax) is widely believed to possess antioxidant and anti-inflammatory properties. In this study, we attempted to evaluate the protective effects of Ax on dextran sodium sulfate (DSS)-induced colitis in mice and the underlying molecular mechanism. Our results suggested that Ax significantly reduced the severity of DSS-induced colitis in mice, as evidenced by increased colon length, decreased disease activity index (DAI), and attenuated inflammatory factors. In addition, Ax significantly increased the diversity of gut microbiota in mice with colitis, remodeled the microbial composition, promoted the production of beneficial bacteria (e.g., Lactobacillaceae), and inhibited the production of harmful bacteria (e.g., Lachnospiraceae and Muribaculaceae). In conclusion, Ax alleviated DSS-induced colitis by maintaining the intestinal barrier and regulating intestinal microbes.

## 1. Introduction

Chronic, recurrent inflammatory bowel disease (IBD) manifests primarily in the gastrointestinal tract and is comprised mainly of two types: Crohn's disease (CD) and ulcerative colitis (UC) [1]. UC tends to be the most prevalent subtype of IBD and is typically marked by ulcerations, bleeding, as well as inflammatory infiltration affecting the mucosa and submucosa of the colon [2]. Recent research indicates that the onset and progression of UC are intricately linked to a severe inflammatory response, the destruction of the intestinal epithelial barrier, and an imbalance in gut flora [3]. Presently, the treatment options available for individuals with UC are quite limited. Frequently used medications, like 5-aminosalicylic acid (5-ASA) and sulfasalazine (SASP) often come with significant adverse effects, such as elevated blood pressure and headaches [4]. Consequently, the exploration of safer, naturally derived bioactive compounds from food sources may represent a promising alternative approach for alleviating the symptoms of UC.

The functionality of the intestinal epithelial cell barrier is primarily regulated by the tight junction (TJ) that exists between adjacent epithelial cells [5]. This junction is a sophisticated network of proteins, chiefly comprised of members from the ZO-1, Occludin, and Claudin families [6]. Numerous studies indicate that irregular expression of TJ proteins in patients with UC can allow bacteria from the abdominal cavity to penetrate the epithelial barrier and enter the intestine, leading to colitis [7]. Consequently, preserving TJ function presents a viable strategy for alleviating UC symptoms [8]. Furthermore, billions of microbiotas contribute to the preservation of host intestinal homeostasis [9]. Recent research indicates that both the composition and metabolism of intestinal flora are significantly linked to the onset and progression of IBD [10]. For instance, maintaining the balance of intestinal flora is crucial for the proper development of the intestinal mucosal immune system and the integrity of the intestinal epithelial barrier, which helps prevent pathogen invasion [11]. Consequently, an imbalance in intestinal flora can elevate the likelihood of pathogen intrusion, leading to intestinal tissue damage and an associated inflammatory response. Intervention in the gut microbiota has been identified as an effective strategy for UC treatment; for example, Phloridin modifies the gut microbiota in mice by generating short-chain fatty acids, which in turn alleviates enterocolitis [12]. Additionally, oral amygdalin enhances UC condition in a manner dependent on microbiota. As such, the regulation of intestinal flora has emerged as a key approach in the treatment of UC [13].

Astaxanthin (Ax) is a keto carotenoid widely found in many marine organisms, such as salmon, shrimp, crabs, and freshwater algae [14]. Furthermore, it is also widely used in food, cosmetics, health care products, and breeding industries at home and abroad [15]. With more than 10 times the antioxidant power of beta-carotene, Ax is considered a natural “beyond antioxidant” [16]. It plays an important role in antioxidation [17], anti-inflammation [18], anticancer [19], immune regulation and so on [20]. For example, recent studies have shown that dietary Ax inhibited the occurrence of early colon cancer in rats which is induced by 1,2-dimethylhydrazine [21]. Ax inhibited Lipopolysaccharide (LPS)-induced inflammation by inhibiting nuclear migration of the nuclear factor Kappa-B (NF-κB) in Macrophages [22]. However, the effect of Ax on the UC model has not been widely studied. This study aimed to determine whether Ax has an effect on colitis induced in mice by dextran sodium sulfate (DSS) and the mechanism by which it operates.

## 2. Materials and Methods

### 2.1. Animal Experiments

**Figure 1:**
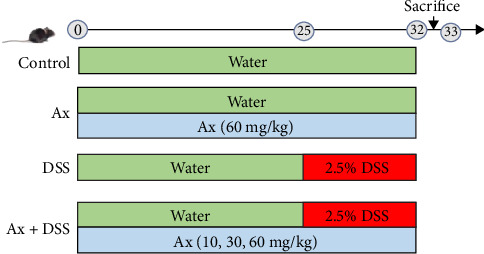
Schematic diagram of the experimental design.

The weight of male C57BL/6J mice, aged 7–8 weeks, was approximately 20 ± 3 g and they were obtained from Liaoning Changsheng Biotechnology Co., Ltd. The mice had unrestricted access to food and water for a week to facilitate adaptation. After this adaptation period, the mice were randomly assigned into six groups (*n* = 5 mice in each group) 1 week later: control group, Ax group (60 mg/kg), DSS (2.5%) group, and DSS + Ax (10, 30, and 60 mg/kg) group. Ax (98%) was purchased from Shanghai Yuanye Biotechnology Co., Ltd. (Shanghai, China) and was gavaged to mice from the first day of the study for 32 days. DSS (2.5% w/v, MP Biomedicals, Santa Ana, USA) was added to drinking water on days 25–32 to induce a mice model of colitis. Mice's body weight was recorded from the time after DSS feeding (Figure 1).

The weight, fecal condition, and diet of the mice were documented daily. By the conclusion of day 32, following the anesthesia and euthanasia of the mice, their colons and feces were collected and preserved at −80°C. All experiments were carried out in accordance with applicable regulations and received approval from the Animal Protection and Use Committee at Changchun University of Traditional Chinese Medicine (located in Changchun, China).

### 2.2. Disease Activity Index (DAI)

The daily monitoring included the body weight and fecal traits of the mice during DSS induction. As previously described [23], DAI was obtained by calculating the arithmetic mean of three scores: weight change, stool consistency, and rectal bleeding.

For the detection of occult blood, a kit from Nanjing Jiancheng Technology Co., Ltd., China, was employed. The methodology for clinical scoring utilized in this research is detailed in Table 1

### 2.3. Histological Analyses

The distal section of the colon was preserved in 4% paraformaldehyde for a period of 24 h. Dehydrated in gradient alcohol and embedded in paraffin wax, and the tissue samples were sectioned to a thickness of 5 µm using a microtome and then stained with hematoxylin–eosin (H&E). The histological alterations were examined using light microscopy.

### 2.4. Detection of Myeloperoxidase (MPO) Activity

A piece of colon tissue was mixed with N-2-hydroxyethyl piperazine-n-ethane-sulfonic acid buffer to make a homogenate and centrifuged at 4°C at 13,000 rpm for 20 min. The resulting precipitate was mixed with 0.5% cetyl trimethyl ammonium chloride. The homogenate was prepared again and the supernatant was collected by centrifugation. The supernatant and color developing solution were added to the 96-well plate for coincubation. After 2 min, sulfuric acid was added to terminate the reaction. The absorbance of the solution is detected at 450 nm, which is the MPO activity.

### 2.5. ELISA

The detection of tumor necrosis factor-alpha (TNF-α), interleukin-1β (IL-1β), and interleukin-6 (IL-6) was performed using ELISA kits (Biolegend, San Diego, CA, USA) following the manufacturer' s guidelines.

### 2.6. Immunofluorescence Staining

The tissue underwent dehydration in a stepwise fashion using ethanol, followed by boiling in sodium citrate buffer. It was then rinsed three times with PBS at room temperature. After sealing with donkey serum and allowing it to sit for 1 h, the tissues were again washed with PBS three times, and subsequently incubated overnight at 4°C with primary antibodies targeting Occludin (1:200; Proteintech, Wuhan, China) and Claudin-3 (1:200; Abcam, Cambridge, UK). Once more, the sections were washed three times with PBS, after which they were treated with goat anti-rabbit IgG (1:1000; Thermo Scientific, China) for 1 h at room temperature. In the final step, the sections received three washes with PBS, and the nuclei were stained with DAPI.

### 2.7. Western Blotting

The extraction of protein was performed using NP40 (Beyotime, Shanghai, China). The protein concentration was assessed utilizing the Pierce BCA protein assay kit (Proteintech, Wuhan, China). A total of 60 μg of colon tissue extract was resolved by sodium dodecyl sulfate–polyacrylamide gel electrophoresis (SDS–PAGE). Following this, the protein was transferred onto a polyvinylidene difluoride (PVDF) membrane (Millipore, Darmstadt, Germany). Subsequently, the membrane was sealed with 5% skimmed milk powder for a duration of 2.5 h. Once sealed, it was incubated overnight at 4°C with primary antibodies targeting Occludin (Proteintech, Wuhan, China), Claudin-3 (Abcam, Cambridge, UK), and β-actin (Proteintech, Wuhan, China). After three washes with TBST, the PVDF membrane was then exposed to goat anti-rabbit IgG (Proteintech, Wuhan, China) at room temperature for 1 h. Finally, a hypersensitive developer is used for detection.

### 2.8. 16S rRNA High Throughput Sequencing

Fecal DNA was extracted and subjected to PCR amplification using primers targeting the hypervariable regions V3–V4 of the 16S rRNA gene (515F and 907R). The amplified PCR products were pooled and purified using the GeneJET Gel Extraction Kit (Thermo Scientific). Sequencing libraries were then prepared and sequenced on the Illumina MiSeq platform, generating 250 bp/300 bp paired-end reads. Sequence data were analyzed using the UPARSE software package (available at http://www.drive5.com/usearch/), employing the UPARSE-OTU and UPARSE-OTUref algorithms. Sequences with ≥97% similarity were grouped into the same OTUs. A representative sequence for each OTU was selected and classification was performed using the RDP classifier to annotate the taxonomic information of each representative sequence. The acquired data is subsequently processed through the cloud platform.

### 2.9. Statistical Analysis

All experiments were performed at least three times independently, with quantitative results expressed as mean values ± SEM based on representative experimental datasets. The GraphPad Prism 10.0.3 was utilized for the data analysis. Image J was utilized for the fluorescent images analysis. Differences between groups were compared by one-way ANOVA and Tukey's multiple comparison test (*p* < 0.05).

## 3. Results

### 3.1. Ax Alleviated the Severity of DSS-Induced Colitis Mice

**Figure 2:**
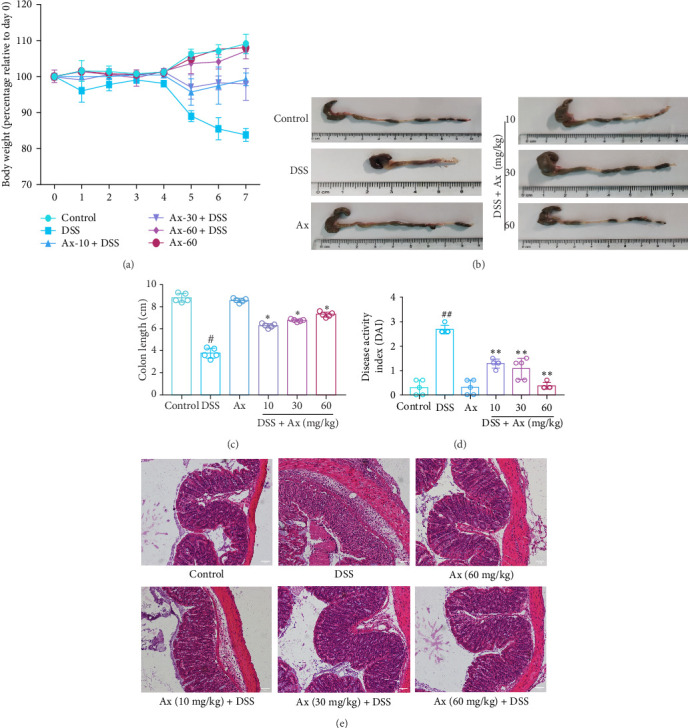
Ax alleviated the severity of DSS-induced colitis mice. (A) Changes in the body weight of the mice were shown. (B, C) Colon length values of mice in each group (*n* = 5 mice per group). (D) Disease activity index (DAI) scores of colitis in each group. (E) Colon tissue samples from each group were stained with H&E (200×). Data are shown as the mean ± SEM (*n* = 3). ^#^p < 0.05 and ^##^*p* < 0.01 compared to the control group; *⁣*^*∗*^*p* < 0.05 and *⁣*^*∗∗*^*p* < 0.01 compared to the DSS group.

To test the protective effect of Ax on DSS-induced colitis in the mice model, we measured weight changes and colon length, and then calculated the DAI. Results demonstrate that in comparison with the control group, the body weight of mice in the DSS model group was significantly reduced, while the body weight of mice after Ax treatment was increased (Figure 2A). Similarly, compared with the control group, mice in the DSS group had a shorter mean colonic length (Figure 2B,C), whereas the Ax-treated mice (60 mg/kg) showed significantly reduced symptoms. DAI scores of each group are shown in Figure 2D. In the DSS group, the DAI score was significantly higher than that in the control group. However, DAI scores decreased significantly after treatment with Ax. Histological changes in the colon were assessed using H&E staining. The H&E staining results revealed that compared with the control group, DSS-induced model mice showed inflammatory cell infiltration, submucosal edema, and crypt structure destruction. In contrast, crypt destruction and epithelial injury were reduced after being treated with Ax (Figure 2E). Taken together, these results suggested that Ax supplementation alleviated symptoms of DSS-induced colitis among mice models.

### 3.2. The Administration of Ax Alleviated the Colonic Inflammatory Response in Mice With DSS-Induced Colitis

**Figure 3:**
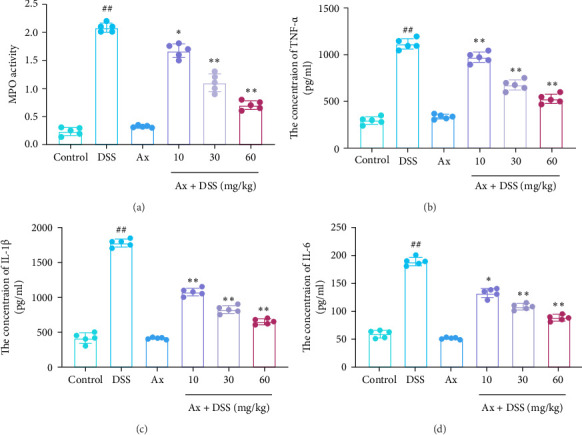
Ax alleviated colonic inflammatory response of DSS-induced colitis mice. (A) MPO activity in colon tissue. The levels of TNF-α (B), IL-1β (C), and IL-6 (D) in colon were detected by ELISA. Data are shown as the mean ± SEM. ^##^*p* < 0.01 compared to the control group; *⁣*^*∗*^*p* < 0.05 and *⁣*^*∗∗*^*p* < 0.01 compared to the DSS group.

It has been reported that MPO is an enzyme that is present in neutrophils and is positively correlated with neutrophil invasion. We found that MPO activity was significantly increased in the DSS group compared with the control group (Figure 3A). In addition, the expression levels of pro-inflammatory cytokines TNF-α (Figure 3B), IL-1β (Figure 3C), and IL-6 (Figure 3D) were significantly decreased after Ax treatment compared with DSS-induced mice. These results support that Ax could alleviate the colonic inflammatory response and immune cell infiltration in DSS-induced colitis mice.

### 3.3. Ax Improved Gut Barrier Integrity in DSS-Induced Colitis Mice

**Figure 4:**
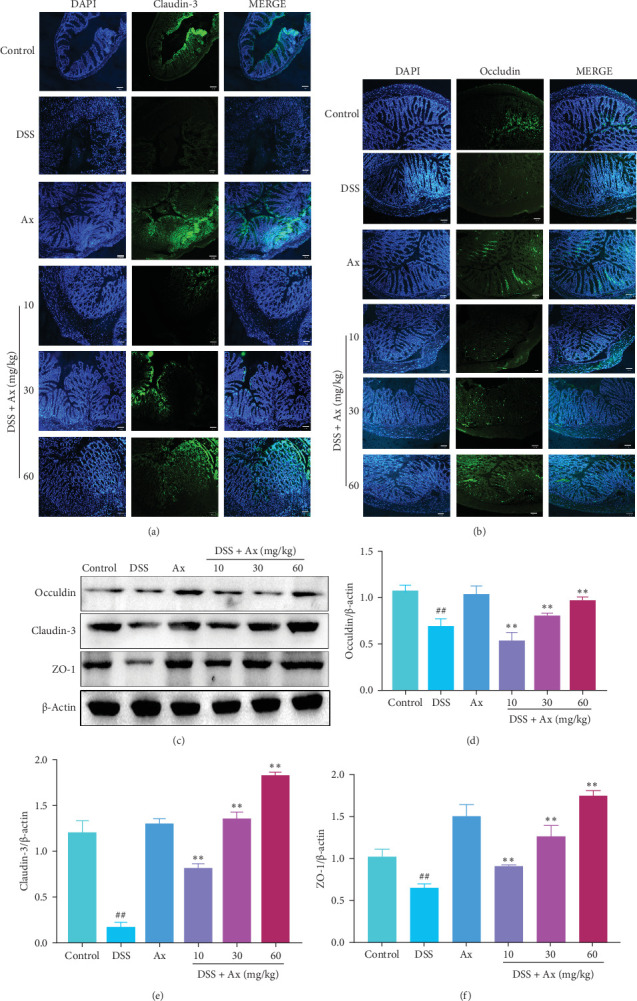
Ax improved gut barrier integrity in DSS-induced colitis mice. The levels of ZO-1 (A) and Occludin (B) in colon tissue were detected by immunofluorescence staining (200×). (C) The levels of Claudin-3, Occludin, and ZO-1 in colon tissues were detected by western blotting. (D) Relative protein abundance of Occludin. (E) Relative protein abundance of claudin-3. (F) Relative protein abundance of ZO-1. Data are shown as the mean ± SEM. ^##^*p* < 0.01 compared to the control group; *⁣*^*∗∗*^*p* < 0.01 compared to the DSS group.

Several previous studies have found that intestinal barrier disruption is the primary pathological feature of colitis and a key contributing factor to IBD [24]. To evaluate the effect of Ax treatment on intestinal TJ proteins, the protein levels of Occludin, Claudin-3, and ZO-1 were detected by performing western blotting and immunofluorescence staining. The western blotting results showed that compared with the control group, the protein levels of Occludin, Claudin-3, and ZO-1 in the DSS group were significantly reduced. However, after being treated with Ax, Occludin (Figure 4C,D), Claudin-3 (Figure 4C,E), and ZO-1 (Figure 4C,F) protein levels in mice were significantly increased. The same results were obtained by immunofluorescence staining (Figure 4A,B). Based on these results, Ax was found to reduce intestinal barrier damage in mice that had been induced with DSS.

### 3.4. Ax Regulates the Diversity of Intestinal Flora in DSS-Induced Colitis Mice

**Figure 5:**
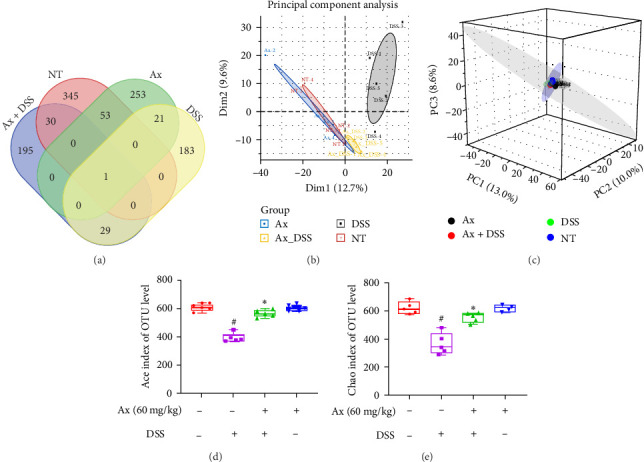
Ax regulated the composition of intestinal microbiota. (A) Venn diagram shows overlapping OTUs in four groups of intestinal flora. (B) PCoA analysis was performed on four groups of samples. Each graph represents a sample. (C) Four groups of samples were analyzed by 3D PCoA. α-diversity was represented by the Chao index (E) and Ace index (D). Data are shown as the mean ± SEM (*n* = 5 mice per group). ^#^*p* < 0.05 compared to the control group; *⁣*^*∗*^*p* < 0.05 compared to the DSS group.

For evaluating the effects of Ax on intestinal flora structure, we have performed 16S rRNA sequencing. Foremost, we identified the common and unique genera of the four taxa (the control group, DSS group, Ax group, and DSS + 60 mg/kg Ax). A Venn diagram shows that 345, 183, 195, and 253 different OTUs were found in the control group, DSS group, Ax + DSS group, and Ax group, respectively, while other OTUs were common (Figure 5A). Next, PCoA analysis has been performed where significant differences were found between the DSS group and control group for intestinal flora where Ax treatment could have ameliorated the changes in intestinal flora among the DSS group (Figure 5B,C). As per α-diversity results, Chao1 (Figure 5E) and Ace (Figure 5D) indices in the DSS group have decreased significantly in comparison to the control group indicating a decrease in flora richness. In contrast, the decrease in bacterial richness index was reversed following Ax treatment.

### 3.5. Ax Treatment Regulated the Composition of Intestinal Microbiota in DSS-Induced Colitis Mice

**Figure 6:**
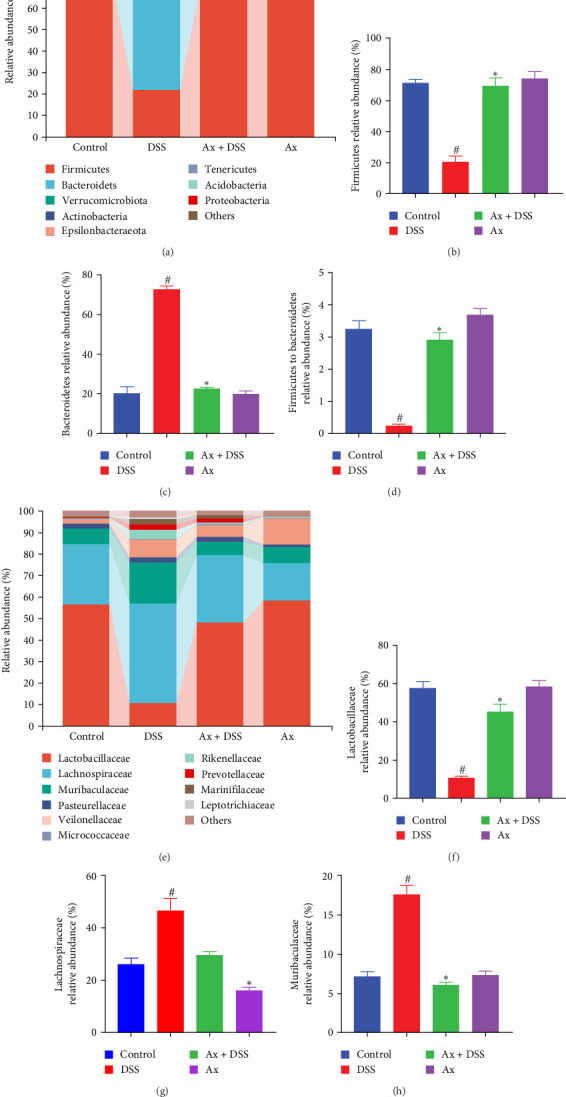
Ax altered the composition of intestinal flora at both phylum and family levels. (A–D) Histogram of relative abundance of dominant bacteria at the phylum level. (E–H) Histogram of relative abundance of dominant fungi at the family level. Data are shown as the mean ± SEM (*n* = 5 mice per group). ^#^*p* < 0.05 compared to the control group; *⁣*^*∗*^*p* < 0.05 to the DSS group.

An analysis of intestinal flora abundance was conducted to determine whether Ax treatment regulates intestinal flora composition. In the control group, *Bacteroidetes* accounted for 20.5% and *Firmicutes* for 72.5%, making them the dominant phyla. Compared to the control, DSS treatment significantly altered the bacterial composition at the phylum level, increasing the abundance of Bacteroidetes and decreasing the abundance of firmicutes. Furthermore, after Ax treatment, the relative abundance of *Firmicutes* increased, while that of *Bacteroidetes* decreased (Figure 6A–C). Additionally, bacterial abundance recovered, and the proportion of *Firmicutes* to *Bacteroidetes* significantly increased following Ax supplementation (Figure 6D).

In the control group, the dominant family were *Lactobacillaceae* (57.7%), *Lachnospiraceae* (25.4%), and *Muribaculaceae* (7.4%). Conversely, DSS treatment significantly altered bacterial composition at the family level, increasing the relative abundance of Trichospiraceae and Muribaculaceae, respectively, while decreasing the relative abundance of Lactobacillaceae. After Ax treatment, the relative abundance of *Lactobacillaceae* increased, while that of *Lachnospiraceae* and *Muribaculaceae* decreased, respectively (Figure 6E–H).

**Figure 7:**
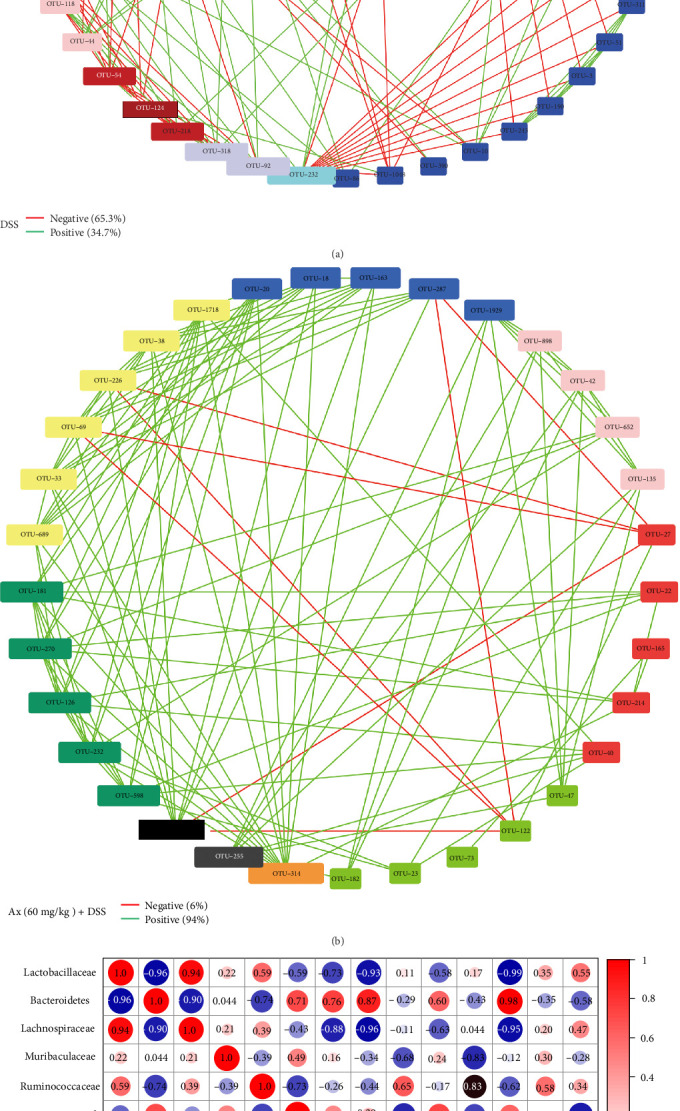
Effects of Ax on dominant microorganisms. (A, B) Intestinal bacterial co-occurrence network structure. Only *p* < 0.05 is displayed with an edge. The degree is the size of the node. (C) Correlation analysis of colon inflammatory cytokines, intestinal barrier, colon length, dominant flora, and MPO. The corresponding numeric value of the correlation index can be found in the box.

Additionally, we analyzed the effect of Ax treatment on bacterial interaction patterns. The core microorganism in the DSS group was *Erysipelotrichaceae* (OTU 232). *Erysipelotrichaceae* showed a positive correlation with *Muribaculaceae* (OTU 68) and a negative correlation with Lactobacillaceae (OUT 17), *Desulfovibrionaceae* (OTU 33), *Lachnospiraceae* (OTU 60), and *Oscillibacter* (OTU 51) (Figure 7A). According to Figure 7B, *Ruminococcaceae* (OTU 314) constituted the core microbe of the Ax + DSS group. *Ruminococcaceae* showed a positive correlation with *Eggerthellaceae* (OUT 126), *Lachnospiraceae* (OTU 270), *Desulfovibrionaceae* (OTU 33), *Clostridiales* (OTU 689), and *Atopobiaceae* (OTU 163). Furthermore, we investigated the correlation between inflammatory markers and various microorganisms. In the network analysis, the *Lactobacillaceae* exhibited a positive correlation with Occludin and a negative correlation with IL-1β, TNF-α, IL-6, the histological score, and the DAI. Conversely, the *Bacteroidetes* demonstrated a positive correlation with IL-1β, TNF-α, IL-6, the histological score, and the DAI, while it showed a negative correlation with Occludin. A negative correlation was observed between the *Lachnospirillaceae* and TNF-α, IL-6, the histological score, and the DAI. The *Muribaculaceae* displayed a negative correlation with MPO and colon length. Additionally, the *Ruminococcaceae* exhibited a negative correlation with IL-1β and the DAI, but it was positively correlated with colon length and Claudin-3 (Figure 7C).

### 3.6. Ax Affects Gene Function in DSS-Induced Colitis Mice

**Figure 8:**
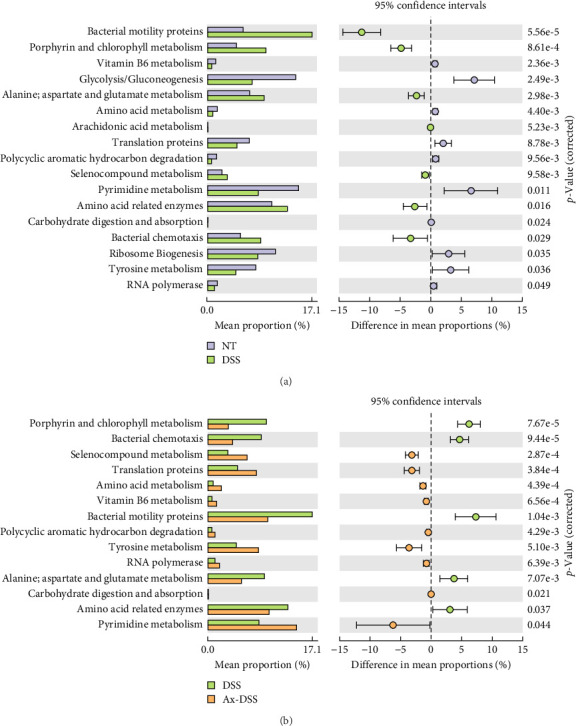
Prediction of the functional content of 16S rRNA gene. (A) KEGG pathway difference between the DSS group and control group, using bilateral Welch's *T* test, *p* < 0.05. (B) KEGG pathway difference between DSS group and the Ax + DSS group was tested by bilateral Welch's *T* test, *p* < 0.05.

A total of 17 KEGG pathways were found to differ between the DSS and control groups, including microbial genes related to carbohydrate metabolism, amino acid metabolism, and vitamin metabolism. In Comparison to the DSS group, Ax treatment restored vitamin B6 metabolism and tyrosine metabolism (Figure 8A,B).

## 4. Discussion

UC is defined by ongoing inflammation of the intestines driven by immune responses, which may lead to symptoms, such as abdominal discomfort, diarrhea, and presence of blood in the stool [25]. The effects caused by DSS in murine models closely resemble those observed in human patients with UC, featuring weight loss, rectal bleeding, and shortened colon lengths [26]. In our research, treatment with Ax resulted in less weight loss, an increase in colon length, and repair of mucosal injuries in mice suffering from DSS-induced colitis.

The integrity and function of the intestinal barrier, along with the interaction between immune cells and intestinal epithelial cells, collaborate to maintain intestinal homeostasis [27]. Consequently, an impaired immune response can disturb gut homeostasis, resulting in chronic and excessive inflammation. Reports indicate that pro-inflammatory cytokines are key pathological elements contributing to inflammation in the intestinal and mucosal regions [24]. Our findings demonstrated that Ax treatment reduced the levels of proinflammatory cytokines (such as IL-6, IL-1β, and TNF-α), which may be related to its improvement of intestinal barrier function and reduction of immune cell overactivation. Furthermore, MPO serves as both a functional and activation marker for neutrophils. In alignment with our perspective, Ax has the capacity to notably reduce MPO activity within colonic tissue.

Occludin and Claudins are integral components of TJs crucial for preserving the integrity of the intestine [27]. Prior research has indicated that UC causes alterations in the tightly linked molecules within the epithelial cells of the colon, resulting in the penetration of bacteria and antigens [28]. Our findings imply that the maintenance of TJ protein expression by Ax in mice is a significant factor in mitigating DSS-induced colitis.

A substantial body of literature indicates that the imbalance of gut microbiota significantly contributes to the advancement of UC [29]. Consistent with these findings, our research demonstrated a decrease in bacterial abundance in the group treated with DSS when compared to the control group. Nonetheless, following treatment with Ax, the α-diversity was restored. Furthermore, distinct clustering patterns were noted between the DSS and Ax groups. The control and Ax groups displayed greater aggregation, in contrast to the DSS group, which deviated from these two. Collectively, these results suggest that the intestinal microflora in the DSS model group underwent considerable alteration in comparison to the control group.

Previous clinical trials have provided evidence indicating that mice with colitis models exhibit a diminished ratio of intestinal flora, specifically *Firmicutes* to *Bacteroidetes* [30] This observation aligns with findings from our study on DSS-induced colitis. Notably, treatment with Ax markedly restored the ratio of *Firmicutes* to *Bacteroidetes*. Certain *Bacteroides* species, for example, have been shown to interfere with the intestinal mechanical barrier. In our investigation, a positive correlation was found between *Bacteroides* levels and DAI, contrasted by a negative correlation with colon length. Additionally, it was noted that the DSS group experienced a significant increase in *Bacteroides*. Conversely, Ax treatment resulted in a substantial decrease in the *Bacteroides* population.

At the family level, *Lactobacilli* are widely recognized as probiotics that contribute to lowering the likelihood of intestinal inflammation [31]. Our findings revealed a negative correlation between *Lactobacilli* and the DAI index, along with inflammatory cytokines (such as IL-6 and TNF-α). Notably, we observed a significant reduction in Lactobacilli within the DSS group. Conversely, treatment with Ax led to a marked increase in the number of *Lactobacilli*. Additionally, it has been established that *Muribaculaceae* are linked to the functionality of the intestinal barrier and the levels of inflammatory cytokines. Our study mirrored these findings, demonstrating a significant rise in *Muribaculaceae* among DSS-treated mice. However, the growth of *Muribaculaceae* was inhibited by Ax treatment. In mouse models of UC, a higher abundance of *Lachnospiraceae* may provoke an exaggerated immune response in the host, worsening the progression of intestinal inflammation [32]. Our results indicated a significant increase in *Lachnospiraceae* in mice suffering from DSS-induced colitis. Nonetheless, Ax treatment notably reduced the levels of *Lachnospiraceae* in the treated mice. These findings suggest that Ax treatment led to a substantial increase in the overall diversity of the flora, an increase in probiotic abundance, and a reduction in the levels of certain pathogenic bacteria when compared to the DSS group.

In addition, research indicates that the interaction patterns of intestinal flora in patients with IBD are disrupted [33]. A notable negative correlation edge was identified within the symbiotic network of DSS-stimulated mice. Conversely, the mice that received Ax treatment demonstrated an enhanced negative correlation edge, implying that Ax therapy may diminish the competitive dynamics among intestinal microorganisms. *Erysipelotrichaceae* is essential within the co-occurrence network observed in DSS-treated groups, and its presence is heightened in individuals with colitis and colorectal cancer. The prominent microorganism found abundantly in the Ax + DSS cohort is *Ruminococcaceae*, known for its anti-inflammatory effects and benefits to gut health. Consequently, treatment with Ax appears to improve the microbial imbalance and fine-tune crucial microbial populations, which could significantly contribute to colitis management.

Additionally, when compared to the DSS group, Ax treatment was found to enhance carbohydrate metabolic pathways that could generate short-chain fatty acids, which then interact with intestinal epithelial cells. Reports indicate a rise in the metabolic pathways associated with vitamin B6, which improves the body's capacity to cope with different adverse situations, including nutritional deficiencies. This study suggests that Ax treatment elevated the metabolism of vitamin B6, potentially bolstering the resistance of mouse colonic epithelial cells to the negative intestinal conditions induced by DSS.

In conclusion, our study demonstrates that Ax treatment significantly alleviates DSS-induced colitis, primarily by reducing MPO and proinflammatory mediators, protecting the intestinal barrier, modulating key gut microbiota, increasing beneficial bacterial populations, and inhibiting harmful bacteria. These findings deepen our understanding of how Ax affects DSS-induced colitis and its mechanisms in regulating immune-related diseases.

The innovative aspect of our study lies in the exploration of Ax's dual effect on both the immune system and gut microbiota. Unlike conventional anti-inflammatory agents that primarily target inflammatory pathways, Ax addresses colitis through a comprehensive strategy, influencing microbial balance, and immune function. This novel mechanism not only enhances our understanding of Ax's role in managing DSS-induced colitis but also provides a promising foundation for future studies investigating its broader therapeutic potential in immune-related diseases. Given the current limitations of existing IBD treatments, these findings underscore the scientific significance of Ax as a promising candidate for the development of new, multitarget therapies.

## Figures and Tables

**Table 1:** Calculated disease activity index (DAI) score.

Score	Weight loss (%)	Stool character	Fecal occult blood
0	0	Normal formed	Negative
1	1–5	Mild loose stool	—
2	5–10	Loose stool	Positive
3	5–10	Mild diarrhea	—
4	>20	Diarrhea	Gross bleeding

## Data Availability

The data will be made available upon request.
